# Supplementary material for: Antioxidant and Lipid-Lowering Effects of Buriti Oil (Mauritia flexuosa L.) Administered to Iron-Overloaded Rats
Source: Molecules. 2023 Mar 13;28(6):2585. doi: 10.3390/molecules28062585 (PMC10056315; doi:10.3390/molecules28062585)
Supplement: Supplementary file 1 [file molecules-28-02585-s001.zip › molecules-2219586-supplementary.pdf]

Supplementary Material

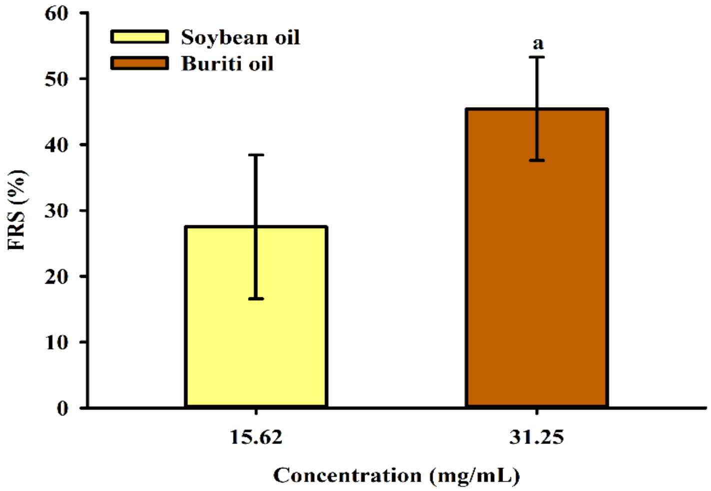

Figure S1. *In vitro* antioxidant potential of soybean and buriti oils.
DPPH= 2,2-diphenyl-1-picrylhydrazyl; FRS= free radicals scavenging. [a] Represent a significant difference between the means ± standard deviations (Student's t-test, $p \leq 0.001$).

Table S1. Diet composition consumed by rats during experimental protocol.

Ingredient	g/100 g diet	Energy (kcal)	
		SC and SFe	BC and BFe
Cornstarch	61.90	247.60	247.60
Casein	14.00	48.00	48.00
Sucrose	10.00	40.00	40.00
Soybean oil	4.00	36.00	–
Buriti oil	4.00	–	36.00
Fiber	05.00	–	–
Mineral mix	3.50	–	–
Vitamin mix	1.00	–	–
DL-methionine	0.30	–	–
Choline bitartrate	0.30	–	–
Total	100.00	341.80	341.80

Diets were prepared following the recommendations of the AIN 93M [1].
SC= control group fed diet containing soybean oil and gavage with saline solution; BC= control group fed diet containing buriti oil and gavage with saline solution; SFe= group fed diet containing soybean oil and gavage with a high-dose $FeSO_4$; BFe= group fed diet containing buriti oil and gavage with a high-dose $FeSO_4$.

Table S2. p-Values of Pearson's correlation matrix.

	Stearic acid	Palmitoleic acid	Oleic acid	Total MUFA	β-Carotene	Tocopherol	Total PUFA	Margaric acid	Miristic acid	Linoleic acid	Total SFA	Linolenic acid	VLDL	TAG	GPx (serum)	Hematocrit	TC
Stearic acid	NA	0.000	0.000	0.000	0.000	0.000	0.001	0.002	0.001	0.001	0.000	0.000	0.963	0.530	0.715	0.747	0.330
Palmitoleic acid	0.000	NA	0.000	0.000	0.000	0.000	0.150	0.075	0.092	0.109	0.209	0.733	0.105	0.864	0.907	0.914	0.382
Oleic acid	0.000	0.000	NA	0.000	0.000	0.000	0.893	0.845	0.920	0.986	0.747	0.216	0.260	0.910	0.829	0.843	0.330
Total MUFA	0.000	0.000	0.000	NA	0.000	0.000	0.824	0.579	0.646	0.706	0.971	0.382	0.212	0.962	0.845	0.858	0.339
β-Carotene	0.000	0.000	0.000	0.000	NA	0.000	0.223	0.376	0.326	0.287	0.159	0.019	0.456	0.758	0.783	0.804	0.317
Tocopherol	0.000	0.000	0.000	0.000	0.000	NA	0.482	0.709	0.639	0.581	0.372	0.069	0.354	0.828	0.804	0.822	0.323
Total PUFA	0.001	0.150	0.893	0.824	0.223	0.482	NA	0.000	0.000	0.000	0.000	0.000	0.087	0.318	0.734	0.780	0.741
Margaric acid	0.002	0.075	0.845	0.579	0.376	0.709	0.000	NA	0.000	0.000	0.000	0.000	0.070	0.326	0.747	0.787	0.802
Miristic acid	0.001	0.092	0.920	0.646	0.326	0.639	0.000	0.000	NA	0.000	0.000	0.000	0.073	0.320	0.744	0.785	0.793
Linoleic acid	0.001	0.109	0.986	0.706	0.287	0.581	0.000	0.000	0.000	NA	0.000	0.000	0.076	0.319	0.742	0.783	0.780
Total SFA	0.000	0.209	0.747	0.971	0.159	0.372	0.000	0.000	0.000	0.000	NA	0.000	0.090	0.322	0.731	0.772	0.731
Linolenic acid	0.000	0.733	0.216	0.382	0.019	0.069	0.000	0.000	0.000	0.000	0.000	NA	0.146	0.324	0.710	0.752	0.617
VLDL	0.963	0.105	0.260	0.212	0.456	0.354	0.087	0.070	0.073	0.076	0.090	0.146	NA	0.000	0.000	0.001	0.011
TAG	0.530	0.864	0.910	0.962	0.758	0.828	0.318	0.326	0.320	0.319	0.322	0.324	0.000	NA	0.000	0.000	0.008
GPx (serum)	0.715	0.907	0.829	0.845	0.783	0.804	0.734	0.747	0.744	0.742	0.731	0.710	0.000	0.000	NA	0.000	0.043
Hematocrit	0.747	0.914	0.843	0.858	0.804	0.822	0.780	0.787	0.785	0.783	0.772	0.752	0.001	0.000	0.000	NA	0.000
TC	0.330	0.382	0.330	0.339	0.317	0.323	0.741	0.802	0.793	0.780	0.731	0.617	0.011	0.008	0.043	0.000	NA

Continuation of Table S2. p-Values of Pearson's correlation matrix.

	Hemo globin	LDL	ALT	AST	Body weight	BMI	Lee index	SOD (serum)	SOD (liver)	WBC	Lymp hocyte s	GPx (liver)	Granul ocytes	Monoc ytes	Platele ts	RBC	Body length	HDL
Stearic acid	0.975	0.000	0.000	0.000	0.179	0.134	0.133	0.372	0.782	0.832	0.809	0.839	0.466	0.100	0.979	0.281	0.503	0.488
Palmitoleic acid	0.497	0.086	0.046	0.039	0.144	0.159	0.202	0.777	0.304	0.735	0.549	0.515	0.509	0.855	0.699	0.506	0.708	0.844
Oleic acid	0.631	0.008	0.005	0.004	0.125	0.116	0.141	0.597	0.396	0.878	0.736	0.602	0.448	0.480	0.766	0.400	0.607	0.693
Total MUFA	0.596	0.015	0.008	0.006	0.127	0.122	0.150	0.634	0.370	0.844	0.690	0.580	0.458	0.555	0.748	0.421	0.627	0.724
β-Carotene	0.750	0.001	0.001	0.001	0.128	0.110	0.126	0.505	0.494	0.981	0.886	0.682	0.431	0.307	0.819	0.355	0.555	0.617
Tocopherol	0.691	0.003	0.002	0.001	0.126	0.112	0.131	0.546	0.444	0.934	0.814	0.646	0.436	0.383	0.792	0.378	0.578	0.650
Total PUFA	0.445	0.008	0.028	0.022	0.798	0.730	0.637	0.438	0.469	0.560	0.359	0.692	0.844	0.080	0.741	0.502	0.643	0.571
Margaric acid	0.429	0.014	0.044	0.035	0.908	0.794	0.692	0.450	0.437	0.541	0.344	0.653	0.872	0.082	0.718	0.552	0.672	0.566
Miristic acid	0.433	0.011	0.038	0.031	0.883	0.784	0.685	0.451	0.447	0.543	0.346	0.653	0.860	0.087	0.733	0.555	0.664	0.575
Linoleic acid	0.437	0.010	0.034	0.027	0.863	0.765	0.668	0.446	0.455	0.545	0.349	0.660	0.852	0.084	0.736	0.544	0.658	0.570
Total SFA	0.458	0.006	0.022	0.017	0.797	0.683	0.592	0.420	0.487	0.552	0.361	0.690	0.816	0.070	0.739	0.500	0.637	0.540
Linolenic acid	0.520	0.002	0.006	0.004	0.612	0.510	0.442	0.380	0.592	0.580	0.405	0.766	0.725	0.058	0.782	0.420	0.584	0.506
VLDL	0.040	0.141	0.851	0.858	0.356	0.284	0.299	0.085	0.000	0.964	0.226	0.457	0.503	0.902	0.816	0.159	0.083	0.244
TAG	0.001	0.075	0.539	0.570	0.994	0.305	0.310	0.165	0.011	0.513	0.028	0.808	0.676	0.448	0.934	0.413	0.030	0.155
GPx (serum)	0.001	0.444	0.862	0.958	0.521	0.028	0.023	0.084	0.093	0.904	0.238	0.081	0.975	0.054	0.766	0.572	0.604	0.017
Hematocrit	0.000	0.025	0.166	0.193	0.183	0.006	0.006	0.575	0.417	0.685	0.478	0.434	0.481	0.099	0.526	0.420	0.126	0.062
TC	0.000	0.260	0.316	0.365	0.000	0.004	0.004	0.936	0.608	0.269	0.980	0.589	0.268	0.044	0.027	0.225	0.427	0.046

Continuation of Table S2. p-Values of Pearson's correlation matrix.

	Hemo globin	LDL	ALT	AST	Body weight	BMI	Lee index	SOD (serum)	SOD (liver)	WBC	Lymp hocyte s	GPx (liver)	Granul ocytes	Monoc ytes	Platele ts	RBC	Body length	HDL
Hemoglobin	NA	0.067	0.111	0.135	0.108	0.027	0.025	0.820	0.958	0.994	0.664	0.309	0.187	0.047	0.159	0.464	0.439	0.048
LDL	0.067	NA	0.000	0.000	0.510	0.267	0.254	0.698	0.501	0.140	0.369	0.111	0.236	0.102	0.802	0.988	0.697	0.705
ALT	0.111	0.000	NA	0.000	0.178	0.148	0.146	0.421	0.064	0.523	0.909	0.380	0.142	0.466	0.059	0.437	0.405	0.133
AST	0.135	0.000	0.000	NA	0.218	0.208	0.206	0.373	0.064	0.547	0.902	0.384	0.144	0.397	0.068	0.423	0.427	0.172
Body weight	0.108	0.510	0.178	0.218	NA	0.000	0.000	0.960	0.751	0.177	0.355	0.654	0.145	0.122	0.059	0.188	0.199	0.091
BMI	0.027	0.267	0.148	0.208	0.000	NA	0.000	0.028	0.890	0.115	0.326	0.811	0.623	0.092	0.024	0.678	0.425	0.001
Lee index	0.025	0.254	0.146	0.206	0.000	0.000	NA	0.025	0.886	0.115	0.324	0.869	0.639	0.077	0.027	0.719	0.426	0.001
SOD (serum)	0.820	0.698	0.421	0.373	0.960	0.028	0.025	NA	0.000	0.854	0.527	0.536	0.031	0.258	0.136	0.540	0.633	0.057
SOD (liver)	0.958	0.501	0.064	0.064	0.751	0.890	0.886	0.000	NA	0.579	0.424	0.359	0.021	0.362	0.880	0.923	0.108	0.455
WBC	0.994	0.140	0.523	0.547	0.177	0.115	0.115	0.854	0.579	NA	0.000	0.478	0.067	0.664	0.799	0.718	0.164	0.105
Lymphocyte s	0.664	0.369	0.909	0.902	0.355	0.326	0.324	0.527	0.424	0.000	NA	0.800	0.054	0.778	0.785	0.761	0.080	0.964
GPx (liver)	0.309	0.111	0.380	0.384	0.654	0.811	0.869	0.536	0.359	0.478	0.800	NA	0.933	0.099	0.959	0.473	0.214	0.503
Granulocyte s	0.187	0.236	0.142	0.144	0.145	0.623	0.639	0.031	0.021	0.067	0.054	0.933	NA	0.715	0.120	0.604	0.065	0.854
Monocytes	0.047	0.102	0.466	0.397	0.122	0.092	0.077	0.258	0.362	0.664	0.778	0.099	0.715	NA	0.266	0.632	0.052	0.035
Platelets	0.159	0.802	0.059	0.068	0.059	0.024	0.027	0.136	0.880	0.799	0.785	0.959	0.120	0.266	NA	0.145	0.309	0.066
RBC	0.464	0.988	0.437	0.423	0.188	0.678	0.719	0.540	0.923	0.718	0.761	0.473	0.604	0.632	0.145	NA	0.229	0.527
Body length	0.439	0.697	0.405	0.427	0.199	0.425	0.426	0.633	0.108	0.164	0.080	0.214	0.065	0.052	0.309	0.229	NA	0.044
HDL	0.048	0.705	0.133	0.172	0.091	0.001	0.001	0.057	0.455	0.105	0.964	0.503	0.854	0.035	0.066	0.527	0.044	NA

NA = not available.

Reference

1. Reeves, P.G.; Nielsen, F.H.; Fahey Jr., G.C. AIN-93 purified diets for laboratory rodents: final report of the American Institute of Nutrition ad hoc writing committee on the reformulation of the AIN-76A rodent diet. *J Nutr* **1993**, *123*, 1939, doi:10.1093/jn/123.11.1939.
